# Extending UTAUT with national identity and fairness to understand user adoption of DCEP in China

**DOI:** 10.1038/s41598-022-10927-0

**Published:** 2022-04-27

**Authors:** Bingyan Wu, Xiaoqing An, Cong Wang, Ho Young Shin

**Affiliations:** 1 School of Economics, Jinan University, Guangzhou City, 510632 Guangdong Province China; 2 School of Business, Yeungnam University, Gyeongsan, Korea

**Keywords:** Engineering, Mathematics and computing

## Abstract

The introduction of digital currency electronic payment (DCEP) by the Central Bank of China is conducive to the central bank's timely grasp of macroeconomic dynamics and the internationalization of RMB. As DCEP is one of the first digital currencies issued by the central bank to be used on a large scale internationally, it is necessary to conduct research on its user adoption. Therefore, this research extends the unified theory of acceptance and use of technology (UTAUT) to explore factors affecting the adoption of DCEP. The researchers cooperated with city banks that have started to use DCEP, and distributed questionnaires to users in the lobbies of these banks. A total of 295 valid questionnaires were empirically examined with Smart-PLS. The results indicate that perceived fairness, habits, social influence and national identity have significant effects on usage, with p values less than 0.05. National identity is shown to be a significant moderator of the relationships between perceived fairness, habit, perceived risk and usage, with p values less than 0.05. National identity is shown to have no moderating effect between social influence and usage, with a p value greater than 0.05. This research provides the central bank and the government with suggestions to increase user enthusiasm and reduce user perceived risks, thereby promoting the widespread use of DCEP.

## Introduction

Digital currency electronic payment (DCEP) is a digitalized version of RMB and a centralized, legal currency. It is issued by the Central Bank of China, operated by designated state-owned banks and freely convertible to the public. Because DCEP has the credit endorsement of the Chinese government, it is beneficial in terms of future RMB settlement, anti-money laundering and internationalization. The large-scale use of DCEP may become a game changer for the global currency system^1^. In contrast to Libra, which is issued by Facebook, DCEP is issued by China and endorsed by national credit, so it involves almost no credit risk, whereas Libra is affected by changes in exchange rates between reserve asset currencies and market confidence. From a regulatory perspective, Libra will eventually be completely decentralized, making it difficult to prevent illegal behaviours such as money laundering and tax evasion. The unrestricted cross-border flow of Libra, which has a strong user base, may weaken the country's currency sovereignty. DCEP transactions are completed at the central bank without any other intermediaries, so the speed of these transactions is much higher than that of e-payment transactions, especially with the spread of 5G networks. When the network is interrupted, DCEP can make payments without it, which is also a feature making DCEP superior to electronic payments.

Since the collapse of the Bretton Woods system, currency issuance has been based on national credit, and the United States, with its strong economic foundation, made the US dollar the world's main reserve currency. In the context of a high degree of globalization, the economic activities of the United States have profoundly affected the economic changes of other countries. Therefore, the current three major financial systems, namely, SWIFT, CHIPS, and Fedwire, are all dominated by the US dollar economy. Regardless of whether they are conducted using US dollars, euros, yen or RMB, cross-border transactions cannot avoid the supervision of the US government, which has raised many concerns among foreign companies about sanctions. Although some nations’ central bank digital currency programs have proven unsuccessful^2,3^, at present, China, the European Union, Japan, Russia and other countries are exploring the construction of their own digital currency payment networks. Another reason that central banks in many countries want to set up their own digital currencies is that they aim to increase consumption and GDP^4–6^.

To protect the interests of commercial banks^7^, a two-tier operating system has been adopted for the central bank’s digital currency. The central bank releases this digital currency through commercial banks, and these commercial institutions then exchange the digital currency with the public. This behaviour not only makes full use of the mature, complete service systems of commercial banks but also prevents direct competition between the central bank and commercial banks. Although there have been some studies on DCEP, most such works focus on its impact on the macro economy, and few studies have focused on the public's acceptance of this currency. If the government wants to actively increase the public's adoption of DCEP to achieve the central bank's goal, the factors that affect the public's usage of the currency must be carefully considered.

Since the Chinese government has many investment projects in Africa, Southeast Asia and South America, it must use foreign exchange regularly and on a large scale. Therefore, problems such as exchange rate loss, payment security, time losses in cross-border remittances and cumbersome procedures lead to massive losses every year. DCEP is intended to improve the efficiency of payments and settlements and greatly reduce transaction costs, which is conducive to promoting the vitality of China's domestic and cross-border trade and finance and increasing commercial value for China. Although DCEP is controlled by the Central Bank of China, the four major state-owned commercial banks (China Industrial and Commercial Bank, China Agricultural Bank, Bank of China, and China Construction Bank), and three communications companies (China Mobile, China Telecom, and China Unicom), people are very worried about their own financial and consumer privacy being completely controlled by the central bank; therefore, some users are cautious about using DCEP. When domestic companies and consumers in China accept DCEP and are willing to use it, they will take DCEP abroad and promote DCEP it throughout the world. Since the Chinese government only issues DCEP in some core cities, and due to the closure caused by the epidemic, it is difficult for foreign researchers to obtain the operation of DCEP. The European Union's digital currency named EUROchain anonymizes customers' identities and transaction records. Except for the intermediaries formulated by the customers, the central bank and other intermediaries cannot see the data submitted by customers. EUROchain can improve customer privacy needs, reduce transaction costs and anti-money laundering. DCEP is an alternative to paper currency issued by the central bank and has the right to monitor all transaction information. Thus, this research also attempts to provide researchers around the world with a thorough understanding of the Chinese people's acceptance of DCEP, as well as the steps taken by the Chinese government to try to counter the US dollar and Euro through DCEP. Therefore, the main purpose and research objective of this paper is to conduct empirical research based on the extended unified theory of acceptance and use of technology (UTAUT) and construct a theoretical model involving consumers in cities that have begun to use DCEP to provide an effective empirical reference for the Chinese central bank for their future domestic and cross-border DCEP payment decisions.

In this study, empirical research is conducted on users of DCEP to identify the factors that influence the public's acceptance of this currency. The structure of the paper is as follows. The introduction discusses the importance of promoting DCEP in terms of enhancing the global competitiveness of China’s central bank and the difficulties faced in this context, and it introduces an empirical research program intended to help the People's Bank of China address the difficulties it encounters in the promotion of DCEP. The literature review exemplifies the popularity of the UTAUT model in scientific research and shows that the PPM model can help improve the effectiveness of empirical research. The methodology section provides not only the research hypotheses and research model, which are constructed by combining UTAUT theory and PPM theory, but also the analysis software selection and basic information on the survey. The results section provides the results of the reliability, validity and hypothesis tests performed. The discussion section provides suggestions for the Central Bank of China in relation to further implementing DCEP in China and contributes to improving China's competitiveness in the context of international finance based on empirical results. The conclusion suggests specific actions to improve the use of DCEP, providing the Central Bank of China with a reference for the formulation of policies. The limitations of this study and future research directions are also given.

## Literature review

UTAUT contains four core dimensions. Performance expectancy refers to the degree to which an individual feels that using the focal system is helpful in their work. Effort expectancy refers to the amount of effort required from an individual to use the system. Social influence refers to the degree to which an individual feels influenced by the group of people around him. Facilitating conditions refer to the degree to which individuals feel that their organizations support their use of the system in terms of related technologies and equipment. UTAUT also recommends some control variables^8^. Researchers can select appropriate variables according to the characteristics of their research objects to add new variables that better explain their research models. When conducting empirical research and testing, researchers often face many theories, and it is difficult to choose one to employ; moreover, they are often forced to choose characteristic variables from among a variety of contradictory models and theories. To respond to this dilemma and unify the literature related to the willingness to accept new technology systems, Venkatesh et al.^8^ proposed UTAUT2 by adding more variables, including habits, price value, hedonic motivation, age, gender and experience, to UTAUT^9^. The ability of UTAUT2 to interpret users' adoption behaviours is much better than that of the UTAUT model. Although UTAUT currently has one of the highest explanatory capabilities for user intentions and usage behaviours among the existing models, many researchers still extend UTAUT to better explain specific user behaviours in different research situations. Table 1 gives a summary of some previous studies that have extended UTAUT to better explain their research objects. By referring to other previous studies, we can provide justification for our methodological choice and enrich our theoretical framework.

**Table 1:** Previous researches extending UTAUT.

Authors	Extended factors	Research object
Alalwan et al.^10^	Trust	Mobile banking
Sheikh et al.^11^	Support, constructs, uncertainty	Social commerce
Reyes-Menendez et al.^12^	Willingness to pay, service quality	Customer loyalty
Reyes-Menendez et al.^13^	Trust, hedonic motivation	Water management promotion
Shaw and Sergueeva^14^	Perceived value	Mobile commerce
Sun et al.^15^	Risk, asset value	Investment in Cambodia
Merhi et al.^16^	Security, Privacy, Trust	Mobile banking
Kapser and Abdelrahman^17^	Risks	Last-mile delivery
Oliveira et al.^18^	Trust, attitude	Collaborative consumption platforms
Gansser and Reich^19^	Security, innovativeness	Artificial intelligence
Kapser et al.^20^	Trust, risk, price sensitivity, innovativeness	Autonomous delivery vehicles
Reyes-Menendez et al.^21^	Social image, perceived product quality	Luxury brands consumption

Because DCEP is issued by the central bank, it differs in specific ways from other technologies and products. First, since the use of DCEP has a certain legal implication stemming from the government, the use of DCEP may not entail sufficient hedonic motivation. Second, DCEP is the digital form of RMB, so it does not have the speculative value of Bitcoin. Third, because users need certain skills related to operating electronic equipment to be proficient in using DCEP, users who are not proficient in operating mobile phones may experience difficulties. Although the Central Bank of China and the government have implemented incentives to encourage the use of DCEP, such as giving free DCEP distributions to citizens to increase their enthusiasm, some citizens still have a negative attitude towards DCEP. To better explore the factors that promote and hinder users from using DCEP, this research introduces a method that combines push-pull-mooring theory and UTAUT.

Push-pull-mooring theory (PPM hereafter) is the most important theoretical paradigm for studying population mobility and immigration, and it is often used as an analytical framework for population migration decision-making. The migration decisions made by populations are the result of the interaction of forces from two different directions: one force promotes migration and is called a push, and the other hinders migration and is called a pull. Between these pushes and pulls, there is a moderating factor called mooring. The pushing factor mainly comprises the internal influencing factors that prompt individuals to temporarily leave their places of residence and go to other destinations, while the pulling factor comprises the external influencing factors that encourage individuals to move. Together, the two forces form the motivation underlying population migration. Usually, the pushing factors impacting immigration behaviour include the depletion of natural resources, increased agricultural production costs, unemployment, lower economic income levels and other negative factors. The pulling factors may include greater employment opportunities, higher salaries, higher living standards, better educational opportunities, more complete cultural facilities, better traffic conditions, a more suitable climate and other positive factors. Mooring factors are factors that moderate immigrant behaviour and generally include innovation ability, identity and belonging, cultural and ethnic diet, and emotional commitment. Zhou^22^ examines users’ switching behaviour between mobile stores using PPM. Fang and Tang^23^ explore involuntary migration in cyberspaces using PPM. Jung et al.^24^ test travellers' switching behaviour in the airline industry from a PPM perspective. Li and Ku^25^ use PPM theory to explain factors affecting e-commerce to social commerce switching. Sun et al.^26^ apply PPM to test SMEs’ adoption of block-chain-based loan systems. PPM is a useful supplement to other theories, such as UTAUT, because pulling variables can explain factors that encourage users to adopt DCEP, pushing variables could explain the factors that hinder users' enthusiasm for using DCEP, and mooring variables may explain the factors that moderate these pulling and pushing effects.

## Methodology

### Pulling effects

The ability of individuals to acquire knowledge is weaker than that of institutions. Institutions can acquire new knowledge rapidly through paid channels, or they can acquire relatively precise knowledge from replicated information by hiring professionals. Therefore, there is a knowledge gap between individuals and institutions. An important feature of the big data era is that commercial organizations can comprehensively collect and analyse consumers’ payment information and data in real time; thus, these organizations can target and accurately market to consumers according to their different preferences, interests, and buying habits. By using data mining and other technologies to predict the upcoming or deep needs of consumers early, institutions can ultimately achieve the great goal of increasing profits. However, because businesses have strong technological monopoly capabilities, consumers often find that unless they no longer use electronic payments, it is difficult to prevent others from tracking their consumption data. The asymmetry of this technology makes consumers unable to deny commercial organizations access to their consumption data even if they feel such access is unfair. DCEP is a payment method that eliminates third-party payments through intermediaries. All its transaction information exists only in the central bank's database, so commercial institutions can no longer obtain information such as payment monitoring data from consumers. Thus, perceived fairness is one of the most important requirements from the perspective of users. In fact, the application of DCEP makes it difficult for government officials to transfer funds obtained by bribery to foreign countries or to use such funds for consumption. This is because any income that enters a personal account from an unidentified source in the form of DCEP without a valid reason may be detected by the central bank. If DCEP can restrain the bribery behaviour of government officials, the public will be satisfied with the government’s integrity; in this way, DCEP can enhance the public’s sense of fairness. Many previous studies have proven fairness is a significant factor for usage in different research fields, including management^27^, tax compliance^28^, regulatory policy^29^, children's behaviour^30^, market power^31^, error management training^32^, reviewer reputation^33^ and contractors’ potential to dispute^34^. Therefore, we propose the following:

#### H1

Perceived fairness positively affects the usage of DCEP.

Habit is a key factor affecting usage behaviour in the context of UTUAT2; this factor refers to an outcome of users’ past behaviours or experiences^8^. Unlike existing third-party payments, DCEP payments do not need to be bound to a bank card, and they are made through only a digital wallet. The use of a DCEP digital wallet makes consumers gradually get used to this new payment method through learning and practice. Despite the government’s efforts, DCEP faces competition from third-party payments such as WeChat and Alipay. WeChat and Alipay have many hundreds of millions of users, and these users have used the payment platforms for years. Since the use of DCEP's e-wallet is not particularly different from that of existing third-party payment platforms, hopefully, users can form DCEP consumption habits within a few years. Habit has been proven to be a significant factor in various research fields, such as those of novel mobile adoption^35^, mapping app usage^36^ and restaurant app usage^37^. Therefore, we propose the following:

#### H2

Habit positively affects the usage of DCEP.

The Chinese government is actively promoting the benefits of DCEP via various media, and these benefits include but are not limited to the ability to make payments without the internet, the ability to avoid payment tracking, anti-money laundering mechanisms, anti-tax evasion mechanisms, anti-telecom fraud mechanisms, and anti-terrorist financing mechanisms. To increase the social influences encouraging the public to adopt DCEP, the Chinese government is also promoting it through administrative channels. In some cities, the salaries and subsidies provided to civil servants are being distributed through DCEP. Additionally, the government requires businesses in some cities to accept DCEP payments. Since there are millions of civil servants in China, the public is bound to be influenced by these policies. When users find that an increasing number of people around them are using DCEP and enjoying its convenience, they may also try to use it. Social influence has been frequently used as a factor in adoption studies, including those on mobile payments^38^, technology adoption by older individuals^39^ and mobile shopping^40^. Therefore, we propose the following:

#### H3

Social influence positively affects the usage of DCEP.

### Pushing effect

Although DCEP has many advantages, consumers will find some potential risks. All the information associated with a payment, whether it is individual or institutional in nature, is obtained by the central bank. Not all institutions and individuals are familiar with tax policies. If the central bank strictly evaluates the tax information of individuals and institutions based on their payment information, large-scale tax payments and fines may ensue. Through the monitoring of DCEP payment records, the government can obtain various types of personal, private information, including health care, insurance, and travel information. With the development of the economy, Chinese users increasingly value the protection of personal privacy, and the risk of private data being monitored may hinder the promotion of DCEP. The negative effect of perceived risk is often tested in various studies, including those on e-store adoption^41^, hotel service adoption^42^, online payments^43^ and consumer reactions^44^. Therefore, we propose the following:

#### H4

Perceived risk negatively affects the usage of DCEP.

### Mooring effect

Most consumers realize that they belong to a country and may express this belonging to the outside world during the consumption process by exhibiting different attitudes towards products from different countries^45^. National identity provides a powerful means by which an individual can be defined and positioned in the world, and it enables people to understand who they are in their globalized and complex environment^46^. Consumers like to signify their identity by consuming iconic national products^47^. Considering that DCEP is used to promote the globalization of China's economy, it is clear that DCEP is a typical iconic national product. National identity has been considered in a wide range of research contexts, including national identity in the United States^48,49^, national identity in New Zealand^50^, national identity in Singapore^51^ and national identity in postcolonial destinations^52^.

The international promotion of DCEP will bring more investment and trade opportunities to China in addition to promoting domestic employment and sustainable corporate development. Currently, the SWIFT system is still the main payment and settlement system used by most banks worldwide. The leadership of the United States is obvious and powerful, which makes its opponents worried that they may be prohibited from conducting any transactions and international trade related to the US dollar. Therefore, the globalization of the RMB is the core of China's economic interests. DCEP is a possible alternative to SWIFT due to its low cost, traceability and reliability. If it is widely used and adopted on a global scale, China's economy will experience unprecedented development. The Chinese government continues to promote the use of DCEP as a patriotic behaviour, hoping to promote the acceptance of DCEP by increasing a sense of national identity among businesses and individuals. When users’ national identities are enhanced, poor people, rich people and businesses may all feel that they can help China's economic globalization process by using this currency. A strong sense of national identity may also encourage users to form the habit of using DCEP more actively. When this sense of national identity forms a culture in Chinese society, users are more likely to be influenced and more inclined to accept DCEP. To enhance China's competitiveness in the global financial market, users with higher levels of national identity may also have reduced perceptions of risk and actively adopt DCEP. Therefore, we propose the following:

#### H5

National identity positively affects the usage of DCEP.

#### H6

National identity positively moderates the relationship between perceived fairness and the usage of DCEP.

#### H7

National identity positively moderates the relationship between habit and the usage of DCEP.

#### H8

National identity positively moderates the relationship between social influence and the usage of DCEP.

#### H9

National identity negatively moderates the relationship between perceived risk and the usage of DCEP.

**Figure 1:**
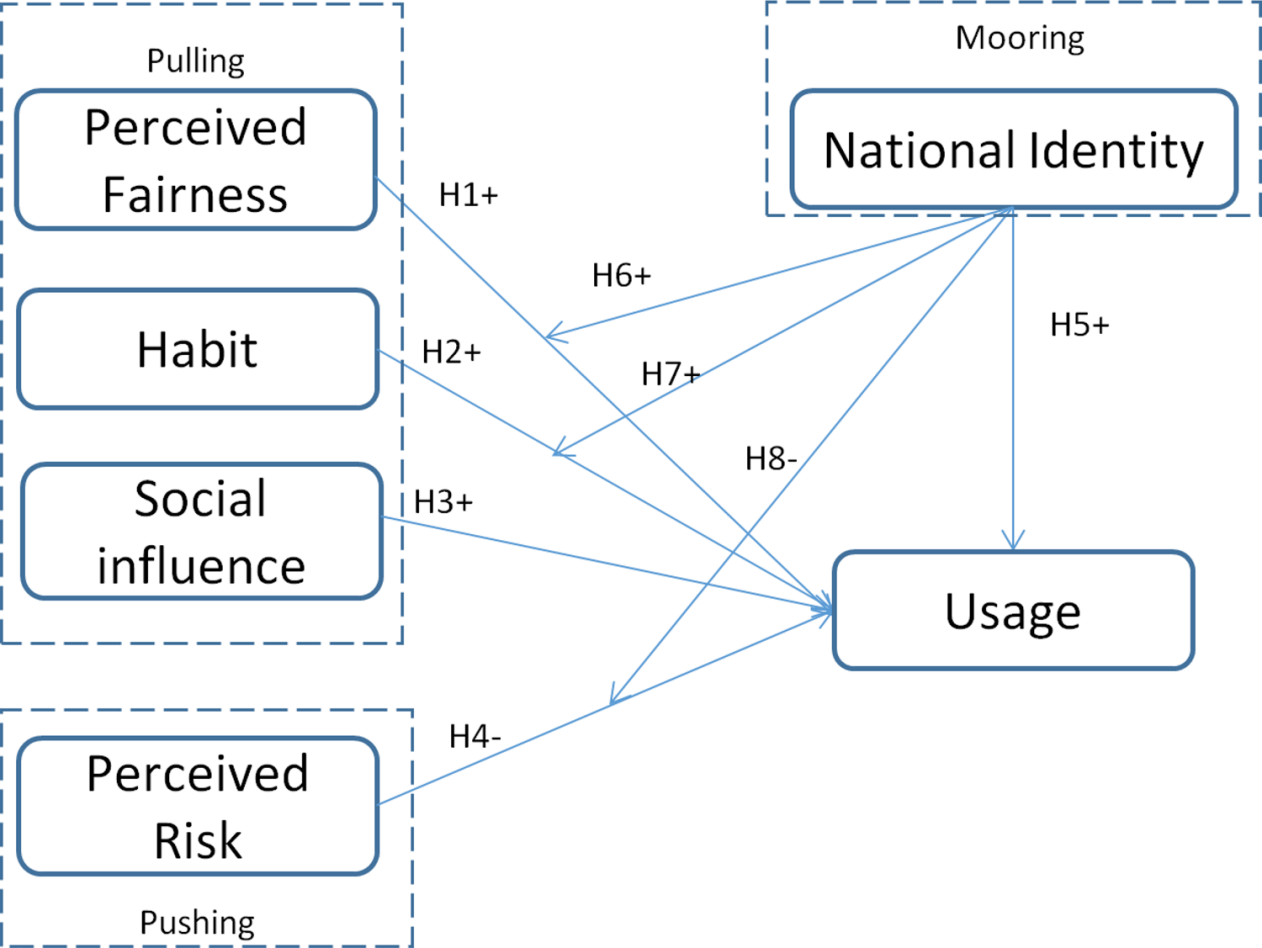
Research model.

Figure 1 depicts all the hypotheses in the research model.

### Survey

All the procedures of this research were carried out in accordance with the relevant guidelines and regulations. All the experimental protocols, including those involving all kinds of ethical behaviours, were approved by the Ethics Commission of Jinan University. Informed consent was obtained from all the participating subjects and/or their legal guardians. The Ethics Commission of Jinan University approved all the experiments, including any relevant details.

The questionnaire was designed to survey DCEP users in China. We cooperated with banks in cities that had started to use DCEP and issued questionnaires to users in the lobbies of these banks; users who voluntarily completed the questionnaires could obtain movie ticket coupons. Most of the DCEP users were civil servants, professionals and businesspeople. They participated voluntarily and authorized the researchers to disclose the survey content to the public. Most of them were under the age of 50 and had a bachelor's degree or above. Most of them had used DCEP for several months for daily consumption purposes. We conducted a pre-test survey of 47 volunteers to improve the reliability and validity of the survey. According to the pre-test results, we improved the questionnaire and then launched a formal online version in June 2021. A total of 341 completed questionnaires were received within two weeks for a completion rate of 67.3%. After deleting any invalid questionnaires, we used 295 valid questionnaires for the empirical test, as shown in Table 2.

**Table 2:** Demographic statistics.

Category	Subject	N	%
Gender	Male	175	59.3%
	Female	120	40.7%
Education level	High school	56	18.9%
	Bachelor	204	69.1%
	Master	31	10.5%
	Ph.D.	4	1.5%
Age	20–30	95	32.2%
	31–40	121	41.0%
	41–50	69	23.3%
	More than 50	10	3.5%
Yearly income	< 50,000$	27	9.1%
	50,000-100,000$	99	33.5%
	100,000–200,000$	112	37.9%
	> 200,000$	57	19.5%
Term of using DCEP	<1month	55	18.6%
	1–3 months	108	36.6%
	3–6 months	126	42.7%
	> 6 months	6	2.1%
Occupation	Civil servants	138	46.7%
	Professionals	83	28.1%
	Businessman	56	18.9%
	Others	18	6.3%

Three items were used to measure perceived risk based on Wu et al. (2011)^53^. Three items were used to measure habits based on Saumell et al.^37^. Three items were used to measure perceived fairness based on Maqsoom et al.^34^. Three items were used to measure social influence based on Grob^40^. Three items were used to measure national identity based on Zhang et al.’s^52^ study. Three items were employed to measure usage based on Sun et al.^54^.

A seven-point Likert scale was used in the measurement process. Smart-PLS 3.0 was used as the main analysis software because DECP is a new technology. Previous researchers have proven that PLS technology provides some advantages related to testing new technology adoption by optimizing restrictive distributions and potential structural modelling^54^^55,56^. Table 2 shows the demographic data of the sample, including those pertaining to gender, education level, age, and yearly income^21^.

## Results

Table 3 gives the evaluation of the basic results of the research model. The Cronbach’s alpha value and composite reliability of all variables were higher than the threshold (0.7). The values of AVE are all higher than the threshold (0.5). The standard loadings are all higher than the threshold (0.6). Therefore, results support the research model's convergence validity and reliability.

**Table 3:** Convergent validity, composite reliabilities testing results.

Construct	Item	Standardized loading	AVE	Composite reliability	Cronbach’s α
Perceived fairness	PF1	0.957	0.914	0.970	0.953
	PF2	0.947			
	PF3	0.964			
Habit	H1	0.971	0.944	0.981	0.970
	H2	0.977			
	H3	0.967			
Social influence	SI1	0.963	0.931	0.976	0.963
	SI2	0.964			
	SI3	0.967			
perceived risk	R1	0.896	0.846	0.943	0.955
	R2	0.993			
	R3	0.866			
National identity	NI1	0.985	0.965	0.988	0.982
	NI2	0.980			
	NI3	0.983			
Usage	U1	0.975	0.951	0.983	0.974
	U2	0.978			
	U3	0.973			

*R* perceived risk, *SI* social influence, *PR* perceived fairness, *H* habit, *NI* national identity, *U* usage.

Tables 4 and 5 indicate discriminant validity test progress. Heterotrait-Monotrait Ratio (HTMT) is the ratio of between-trait and within-trait. It is the ratio of the mean value of index correlation between different dimensions to the mean value of index correlation between the same dimensions^57^. All the values in Table 4 are less than the threshold value (0.85). All the values in Table 5 are less than the threshold value (1.0). Therefore, the research model’s discriminant validity is supported.^58^.

**Table 4:** Discriminant validity (Heterotrait–Monotrait ratio).

	NI*H	H	NI	NI*R	SI	NI*SI	U	NI*PR	PR	R
NI*H										
H	0.305									
NI	0.246	0.227								
NI*R	0.454	− 0.188	0.257							
SI	0.109	0.381	0.259	0.165						
NI*SI	0.439	− 0.117	0.230	0.288	0.198					
U	0.007	0.300	0.272	0.232	0.439	0.111				
NI*PR	0.089	0.022	0.253	0.139	0.033	0.195	0.100			
PR	0.025	0.230	0.338	0.022	0.424	0.039	0.309	0.503		
R	0.200	0.188	0.150	0.226	0.124	0.189	0.0088	0.021	0.119	

*R* perceived risk, *SI* social influence, *PR* perceived fairness, *H* habit, *NI* national identity, *U* usage.

**Table 5:** Bootstrapping confidence interval up of HTMT.

NI*H->U	0.275
H->U	0.296
NI->U	0.286
NI*R->U	− 0.044
SI->U	0.381
NI*SI->U	0.015
NI*PR->U	0.260
PR->U	0.337
R->U	− 0.017

*R* perceived risk, *SI* social influence, *PR* perceived fairness, *H* habit, *NI* national identity, *U* usage.

**Figure2:**
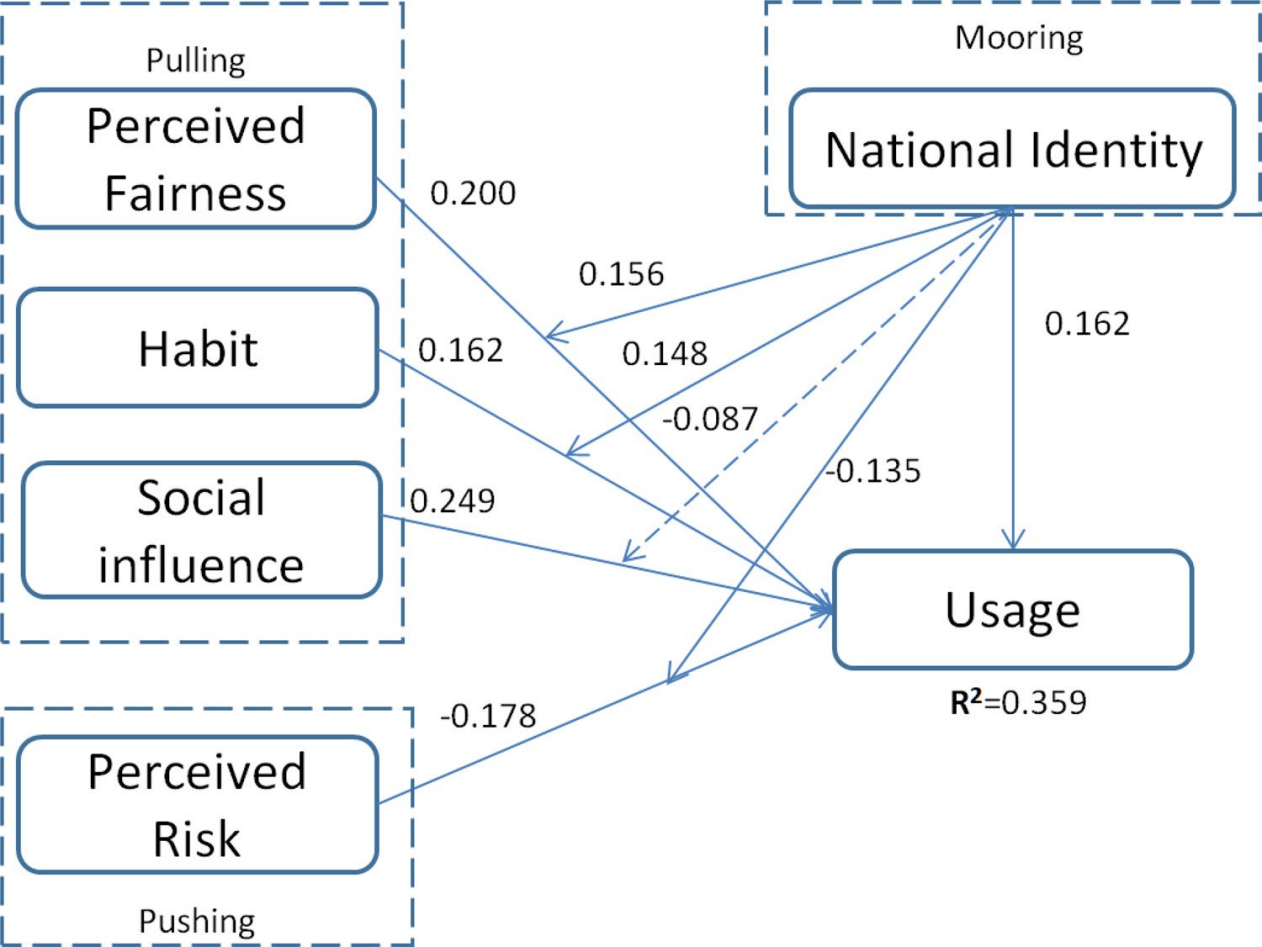
Structural model (P < 0.05).

Figure 2 is the structural model which shows the empirical test results by PLS method. H1, H2, H3, H4, H5, H6 and H7 are supported with P value less than 0.05. R^2^ means how much the independent variables can explain the proportion of usage variation by regression. Based on the structural model, it shows usage can be explained by all the five independent variables in variance proportion of 35.9%^59^.

## Discussion

In this study, social influence is proven to be significantly and positively related to usage, with a p value less than 0.05. Professional services and technical teams are required to expand the social influence of DCEP; however, all the current promoters are banks. Payment companies and services have complete service teams specialized in payments, and the provision of these professional services can help enhance the social influence of DCEP. The Chinese government and central bank should bring together payment companies and service providers to improve the coverage of the last mile of payments. In this context, payment companies and service providers could encourage their customers to obtain the corresponding remuneration while expanding the scope of their own businesses to achieve revenue growth. Habits are proven to be significantly and positively related to usage, with a p value less than 0.05. When users are accustomed to using digital payment methods such as Alipay and WeChat Pay on a daily basis, DCEP is easily accepted and habit-forming. Additionally, perceived risk is proven to be significantly and negatively related to usage^60^, with a p value less than 0.05. The government's use of DCEP reduces the cost of tracking and verifying transactions, and more information is thus revealed about users' tax payments; therefore, users face increased difficulty in relation to tax avoidance. The government should adjust its mechanism of taxation on enterprises and individuals and thereby reduce the intensity of taxation imposed on users, as this could help them maintain survival and profitability from the perspective of taxation. Otherwise, most enterprises and individual users will perceive a potential taxation risk in relation to DCEP and may refuse to adopt it.

National identity is proven to be significantly and positively related to usage with a p value less than 0.05, which indicates that Chinese users proudly believe that DCEP has a global leading edge in the context of technology and that their active use of DCEP can help China take the initiative in international economic competition. The Chinese government and central bank must not only promote the convenience, speed and low cost of DCEP but also explain to users that the true competitors of DCEP are the many digital currencies in the future international market; in this way, users' national identity can be strengthened. It is proven that national identity can positively moderate the relationship between perceived fairness and usage, and this relationship has a p value less than 0.05. The reason for this phenomenon could be that, even when people's wealth and class differ, they are willing to actively use DCEP to clearly demonstrate their national identities. Thus, the government should encourage institutions and individuals to actively use DCEP instead of other currencies in domestic and cross-border transactions to increase the influence of DCEP in the global financial market, which could make RMB receive fairer treatment. It is proven that national identity can positively moderate the relationship between habit and usage with a p value less than 0.05. This could be because the Chinese government's long-term promotion of national identity in the media has made the public more accustomed to accepting new products or technologies on the grounds of safeguarding national interests. Unfortunately, it is proven that national identity cannot moderate the relationship between social influence and usage, and this finding has a p value greater than 0.05. The reason for this could stem from the fact that although many people and media groups emphasize the importance of using DCEP for the good of the country, different people have different opinions. It is proven that national identity can negatively moderate the relationship between perceived risk and usage with a p value less than 0.05. This shows that when people’s national identity is strong, many will actively reduce their perceptions of potential risks and choose to enthusiastically accept DCEP.

These findings reflect the ambition of the Central Bank of China in relation to digital technology and financial globalization. This study provides detailed policy suggestions for the Central Bank of China regarding the further implementation of DCEP in China and contributes to improving the country’s competitiveness in international finance. The study not only provides a reference for other countries to implement their own digital currencies in the future but also helps central banks construct a decision-making basis for promoting tax growth and social justice through digital currency. By extending UTAUT, this study incorporates push-pull theory and the national identity factor into its model and proves their importance as modular variables and moderating factors.

## Conclusions

The results obtained through this empirical research support the hypotheses well and clarify the objectives of the research. The Central Bank of China can refer to these research conclusions to effectively promote DCEP and thus achieve the original purpose of this research. To improve users’ habits, the government can subsidize the pre-installation of DCEP wallets in mobile phones produced in China to promote the public’s habit of paying with DECP. In addition, for Chinese-funded overseas banks, it is recommended that DCEP users are provided with additional comprehensive foreign exchange services in the future. Third-party agencies such as law firms and accounting firms can review DCEP records to understand the true financial situations of companies and issue more accurate professional recommendations, thereby creating a fair, competitive environment for the public in terms of loan interest rates and tax payments. Due to the low cost and high efficiency of DCEP payments, banks can issue DCEP-based credit cards that offer customers’ higher point rewards, lower annual fees, and instalment fee discounts, thereby increasing the social influence of DCEP. The introduction of DCEP overseas could theoretically expand the scope of RMB usage and promote the cross-border settlement of RMB. Potential DCEP users may include trading partners and people in the countries and regions along the “Belt and Road” who do not yet use banking services. The establishment of another international payment system is also expected to reduce reliance on foreign exchange. Users can have DCEP wallets even if they do not have bank accounts in China. If DCEP can be freely converted into non-RMB currencies and transferred abroad, the number of international users of DCEP may increase. Previous researches have demonstrated that the Chinese government is very good at extending its familiar business models to other developing countries^61,62^. To increase fairness, institutions can grant loans in the form of DCEP to facilitate accurate loan usage tracking and prevent misappropriation, thereby greatly reducing the possibility of the falsification of cash flows, assets and liabilities, profit and other data. To reduce the perceived risk of lenders, banks could give preferential interest rates to companies that provide DCEP loans to enhance the popularization of DCEP in the context of financial activities.

Despite the contributions of this research, it also has some limitations. First, because the survey was conducted in a few large cities in China, considering the uneven development of China’s cities, the research conclusions may not be fully applicable to small, remote cities. Second, most of the individuals who responded to the questionnaire were middle-class people; therefore, future research should also focus on low-income groups, such as students, farmers, and freelancers. Finally, this research is mainly aimed at users, and it ignores the role of financial institutions in the promotion of DCEP. Future research can conduct questionnaire surveys among financial institutions to obtain useful suggestions from them (Supplementary Information).

## Supplementary Information

Supplementary Information 1.

Supplementary Information 2.
