# Supplementary material for: Extending UTAUT with national identity and fairness to understand user adoption of DCEP in China
Source: Sci Rep. 2022 Apr 27;12:6856. doi: 10.1038/s41598-022-10927-0 (PMC9046427; doi:10.1038/s41598-022-10927-0)
Supplement: Supplementary file 1 — Supplementary Information 1. [file 41598_2022_10927_MOESM1_ESM.docx]

**Extending UTAUT with National Identity and Fairness to Understand User Adoption of DCEP in China** (09-06-2021)

If you agree, please paste√in the bar of “I agree”

| **Disclaimer** | |
| --- | --- |
| 1. I volunteered to take part in this anonymous survey.  2. All information is authorized to be public.  3. No conflict of interest exists. | I agree______ |

Please paste√ in the bar of your choice

|  | Male | Female |
| --- | --- | --- |
| Gender |  |  |

Please paste√ in the bar of your choice

|  | High School | Bachelor | Master | Ph.D |
| --- | --- | --- | --- | --- |
| Education level |  |  |  |  |

Please paste√ in the bar of your choice

|  | 20-30 | 31-40 | 41-50 | More than 50 |
| --- | --- | --- | --- | --- |
| Age |  |  |  |  |

Please paste√ in the bar of your choice

|  | <50,000 | ≥50,000&<100,000 | ≥100,000&<200,000 | ≥200,000 |
| --- | --- | --- | --- | --- |
| Yearly Income  (us dollar) |  |  |  |  |

Please paste√ in the bar of your choice (month)

|  | <1 | ≥1&<3 | ≥3&<6 | ≥6 |
| --- | --- | --- | --- | --- |
| Period of using DCEP |  |  |  |  |

Please paste√ in the bar of your choice

|  | Civil servants | Professionals | Businessman | Others |
| --- | --- | --- | --- | --- |
| Occupation |  |  |  |  |

Please paste√ in the bar of your choice (1-strongly disagree; 7-strongly agree)

| Perceived fairness | ① | ② | ③ | ④ | ⑤ | ⑥ | ⑦ |
| --- | --- | --- | --- | --- | --- | --- | --- |
| No company or individual can evade taxes by using DCEP makes me feel fair. |  |  |  |  |  |  |  |
| Using DCEP can reduce bribery and insider trading and promote fair competition. |  |  |  |  |  |  |  |
| Using DCEP provides companies or individuals with more opportunities for fair competition. |  |  |  |  |  |  |  |

Please paste√ in the bar of your choice (1-strongly disagree; 7-strongly agree)

| Habit | **①** | **②** | **③** | **④** | **⑤** | **⑥** | **⑦** |
| --- | --- | --- | --- | --- | --- | --- | --- |
| I am in favor of using DCEP. |  |  |  |  |  |  |  |
| I feel the need to use DCEP. |  |  |  |  |  |  |  |
| Using DCEP in daily life has become natural to me. |  |  |  |  |  |  |  |

Please paste√in the bar of your choice (1-strongly disagree; 7-strongly agree)

| Perceived risk | **①** | **②** | **③** | **④** | **⑤** | **⑥** | **⑦** |
| --- | --- | --- | --- | --- | --- | --- | --- |
| Wages and consumption in the form of DCEP will cause a lot of inconvenience. |  |  |  |  |  |  |  |
| It is risky to use DCEP in business transactions and daily consumption. |  |  |  |  |  |  |  |
| Using DCEP may cause me to pay more taxes. |  |  |  |  |  |  |  |

Please paste√in the bar of your choice (1-strongly disagree; 7-strongly agree)

| National identity | **①** | **②** | **③** | **④** | **⑤** | **⑥** | **⑦** |
| --- | --- | --- | --- | --- | --- | --- | --- |
| A strong sense of belonging in China encourages me to use DCEP. |  |  |  |  |  |  |  |
| My Chinese identity makes me confidently to use DCEP. |  |  |  |  |  |  |  |
| I am proud to use DCEP to promote China’s economic development. |  |  |  |  |  |  |  |

Please paste√in the bar of your choice (1-strongly disagree; 7-strongly agree)

| Social Influence | **①** | **②** | **③** | **④** | **⑤** | **⑥** | **⑦** |
| --- | --- | --- | --- | --- | --- | --- | --- |
| My friend suggested that I should try DCEP. |  |  |  |  |  |  |  |
| There are positive reports about using DCEP. |  |  |  |  |  |  |  |
| In general, most people welcome DCEP. |  |  |  |  |  |  |  |

Please paste√in the bar of your choice (1-strongly disagree; 7-strongly agree)

| Usage | **①** | **②** | **③** | **④** | **⑤** | **⑥** | **⑦** |
| --- | --- | --- | --- | --- | --- | --- | --- |
| I am interested in using DCEP. |  |  |  |  |  |  |  |
| In daily consumption, I will pay in DCEP. |  |  |  |  |  |  |  |
| I will recommend DCEP to others. |  |  |  |  |  |  |  |
